# Supplementary material for: Subcellular mass spectrometry imaging of lipids and nucleotides using transmission geometry ambient laser desorption and plasma ionisation
Source: Nat Commun. 2025 Oct 15;16:9130. doi: 10.1038/s41467-025-64604-7 (PMC12528750; doi:10.1038/s41467-025-64604-7)
Supplement: Supplementary file 2 — Reporting Summary [file 41467_2025_64604_MOESM2_ESM.pdf]

nature portfolio

Corresponding author(s): Shane R. Ellis

Last updated by author(s): Sep 11, 2025

Reporting Summary

Nature Portfolio wishes to improve the reproducibility of the work that we publish. This form provides structure for consistency and transparency in reporting. For further information on Nature Portfolio policies, see our Editorial Policies and the Editorial Policy Checklist.

Statistics

For all statistical analyses, confirm that the following items are present in the figure legend, table legend, main text, or Methods section.

n/a	Confirmed	
☐	☒	The exact sample size (n) for each experimental group/condition, given as a discrete number and unit of measurement
☐	☒	A statement on whether measurements were taken from distinct samples or whether the same sample was measured repeatedly
☒	☐	The statistical test(s) used AND whether they are one- or two-sided<br>*Only common tests should be described solely by name; describe more complex techniques in the Methods section.*
☒	☐	A description of all covariates tested
☒	☐	A description of any assumptions or corrections, such as tests of normality and adjustment for multiple comparisons
☒	☐	A full description of the statistical parameters including central tendency (e.g. means) or other basic estimates (e.g. regression coefficient) AND variation (e.g. standard deviation) or associated estimates of uncertainty (e.g. confidence intervals)
☒	☐	For null hypothesis testing, the test statistic (e.g. F, t, r) with confidence intervals, effect sizes, degrees of freedom and P value noted<br>*Give P values as exact values whenever suitable.*
☒	☐	For Bayesian analysis, information on the choice of priors and Markov chain Monte Carlo settings
☒	☐	For hierarchical and complex designs, identification of the appropriate level for tests and full reporting of outcomes
☒	☐	Estimates of effect sizes (e.g. Cohen's d, Pearson's r), indicating how they were calculated

Our web collection on statistics for biologists contains articles on many of the points above.

Software and code

Policy information about availability of computer code

Data collection	MSI data were acquired using a modified Bruker timsTOF Pro mass spectrometer using a custom developed version of Bruker timsControl (v6.0.0-SNAPSHOT 27e73e95) and flexImaging (v7.3 Build 9) that allowed control of the non-standard source stage. IC Capture (v2.5) was used for sample visualisation using the on-board camera optic. Leica LASX Office v1.4.4.26810) was used to obtain brightfield and fluorescence microscopy. FIB-SEM data were collected using ThermoScientific xTm software (v14.5.1.432).
Data analysis	Bruker SCiLS Lab (v2025b Pro) was used for MS image analyses and visualisation of the data. Leica LASX office (v1.4.4.26810) was used for microscopy image visualisation. Microsoft Excel was used for lipid identification and target list generation to be fed back into SCiLS workflows. Microsoft PowerPoint was used for image compilation and figure component creation.

For manuscripts utilizing custom algorithms or software that are central to the research but not yet described in published literature, software must be made available to editors and reviewers. We strongly encourage code deposition in a community repository (e.g. GitHub). See the Nature Portfolio guidelines for submitting code & software for further information.

Data

Policy information about availability of data

All manuscripts must include a data availability statement. This statement should provide the following information, where applicable:

- Accession codes, unique identifiers, or web links for publicly available datasets
- A description of any restrictions on data availability
- For clinical datasets or third party data, please ensure that the statement adheres to our policy

As stated in the manuscript: The data generated in this study have been deposited in the Zenodo database under accession code DOI: 10.5281/zenodo.15477922 or can be obtained from the corresponding author on request. The spectral and graphical data used for figures are provided in the Source Data file.

Research involving human participants, their data, or biological material

Policy information about studies with human participants or human data. See also policy information about sex, gender (identity/presentation), and sexual orientation and race, ethnicity and racism.

Reporting on sex and gender	This information does not pertain to this study.
Reporting on race, ethnicity, or other socially relevant groupings	This information does not pertain to this study.
Population characteristics	This information does not pertain to this study.
Recruitment	This information does not pertain to this study.
Ethics oversight	This information does not pertain to this study.

Note that full information on the approval of the study protocol must also be provided in the manuscript.

Field-specific reporting

Please select the one below that is the best fit for your research. If you are not sure, read the appropriate sections before making your selection.

[x] Life sciences [ ] Behavioural & social sciences [ ] Ecological, evolutionary & environmental sciences

For a reference copy of the document with all sections, see nature.com/documents/nr-reporting-summary-flat.pdf

Life sciences study design

All studies must disclose on these points even when the disclosure is negative.

Sample size	As a methods development project, the focus of this study was in selecting regions of interest from similar tissue areas so as to accurately determine the best sample preparation methods for single cell and subcellular imaging. However, three serial sections of mouse brain were analysed using the various techniques, with 2-3 near-identical regions being selected for each. Depending on the pixel size, MS images consist of 500,000+ individual spectra, which were monitored for inconsistencies in the data. Cells were cultured on chambered glass slides, with each of the 12 chambers containing 10,000-100,000 cells. Regions of interest were selected from each chamber based on confluence, and single cells serve as replicates.
Data exclusions	No data were explicitly excluded, however the lipid identification tool described within the manuscripts methods section does employ signal thresholding and mass accuracy calculations to most accurately identify lipids with the highest possible confidence.
Replication	Numerous MSI were taken from each tissue and cell line, however 1 mm2 images at 1 um pixel sizes take ~35 hours of continuous acquisition so replicates were limited to 3 brain and spinal cord tissues, and 3-4 slides chambers per each cell line. Because the focus of this study was the optimisation of sample preparation, laser optics and laser power regimes for obtaining minimum pixel sizes while maintaining analyte coverage, replicates consist of both similar and dissimilar laser parameters for achieving identical pixel sizes to those represented in the figures and supplementary information. All attempts at replication were successful.
Randomization	Randomization does not pertain to the work conducted within this study.
Blinding	Blinding does not pertain to the work conducted within this study.

Reporting for specific materials, systems and methods

We require information from authors about some types of materials, experimental systems and methods used in many studies. Here, indicate whether each material, system or method listed is relevant to your study. If you are not sure if a list item applies to your research, read the appropriate section before selecting a response.

Materials & experimental systems

n/a	Involved in the study
x	Antibodies
	x Eukaryotic cell lines
x	Palaeontology and archaeology
	x Animals and other organisms
x	Clinical data
x	Dual use research of concern
x	Plants

Methods

n/a	Involved in the study
x	ChIP-seq
x	Flow cytometry
x	MRI-based neuroimaging

Eukaryotic cell lines

Policy information about cell lines and Sex and Gender in Research

Cell line source(s)	SH-SY5Y cells (neuroblastoma; RRID: CVCL_0019), U2OS cells (osterosarcoma; RRID: CVCL_0042) and previously established patient lines (UWG01CTC, UWG02CTC and UWG02ASC) were obtained from culture stocks stored within the University of Wollongong.
Authentication	None of the cell lines used were authenticated.
Mycoplasma contamination	SH-SY5Y, U2OS and UWG lines were not tested for mycoplasma contamination.
Commonly misidentified lines (See ICLAC register)	No commonly misidentified cell lines were used within this study.

Animals and other research organisms

Policy information about studies involving animals; ARRIVE guidelines recommended for reporting animal research, and Sex and Gender in Research

Laboratory animals	Mouse organs were harvested from NSG NOD.Cg-Prkdcscid Il2rgtm1Wjl (brain) and C57BL/6 (spinal cord) mice, produced from other research within the University of Wollongong in accordance with the Australian code for the care and use of animals for scientific purposes. Mice were housed under standard conditions (19–26°C and 40–70% humidity, 12-hour light/dark cycle).
Wild animals	No wild animals were used within this study.
Reporting on sex	Due to this being a method development study, no sex-based information was collected.
Field-collected samples	No field-collected samples were used within this study.
Ethics oversight	All animal experiments were approved by the University of Wollongong Animal Ethics Committee (approval number: AEPR22/17) and complied with the Australian National Health and Medical Research Centre code of practice for the care and use of animals for scientific purposes.

Note that full information on the approval of the study protocol must also be provided in the manuscript.

Plants

Seed stocks	Not applicable.
Novel plant genotypes	Not applicable.
Authentication	Not applicable.
