# Supplementary material for: Subcellular mass spectrometry imaging of lipids and nucleotides using transmission geometry ambient laser desorption and plasma ionisation
Source: Nat Commun. 2025 Oct 15;16:9130. doi: 10.1038/s41467-025-64604-7 (PMC12528750; doi:10.1038/s41467-025-64604-7)
Supplement: Supplementary file 3 — Description of Additional Supplementary Files [file 41467_2025_64604_MOESM3_ESM.pdf]

File name: Supplementary Data 1

Description: Microscopy reporting table, displaying a standardised format of parameters used for acquisition and processing of microscopy images.
